# The Role of High Serum Apelin Levels Within the First 24 h in Predicting 28-Day and Long-Term Mortality in Ischemic Cerebrovascular Disease

**DOI:** 10.3390/jcm15166469

**Published:** 2026-08-21

**Authors:** Kübra Işık, Asım Tekin, Yakup Özer, Burak Mete

**Affiliations:** 1 Department of Neurology, Faculty of Medicine, Zonguldak Bülent Ecevit University, Zonguldak 67020, Turkey; drkbra4406@gmail.com; 2 Department of Internal Medicine, Sanlıurfa Training and Research Hospital, Sanlıurfa 63200, Turkey; tekinasim@hotmail.com (A.T.); yakupozerdr@gmail.com (Y.Ö.); 3 Department of Public Health, Faculty of Medicine, Cukurova University, Adana 01130, Turkey

**Keywords:** apelin, ischemic stroke, mortality, biomarker, prognosis, survival analysis

## Abstract

**Background/Objectives:** The apelin/APJ system has demonstrated neuroprotective effects in experimental studies by regulating key pathogenic mechanisms of ischemic stroke, including oxidative stress, apoptosis, cerebral edema, and inflammation. This study aimed to investigate the role of serum apelin levels measured within the first 24 h in predicting 28-day and long-term mortality in patients with ischemic cerebrovascular disease. **Methods:** This prospective cohort study included 44 patients hospitalized with a diagnosis of acute ischemic stroke due to large artery atherosclerosis. Serum apelin levels were measured using the ELISA method from blood samples obtained within the first 24 h after symptom onset. Patients were prospectively followed for 28-day and long-term mortality. **Results:** During the follow-up period, 32.6% of the patients died. The median serum apelin level was significantly higher in the deceased group compared to the survivors [105.0 pg/mL vs. 55.8 pg/mL]. In ROC analysis, the area under the curve (AUC) for apelin in predicting mortality was 0.727 (95% CI: 0.550–0.903). The optimal cut-off value was determined as ≥70.47 pg/mL (sensitivity: 85.71%, specificity: 62.07%). In Kaplan–Meier analysis, the 28-day mortality rate was 52.2% in the high-apelin group (≥70.47 pg/mL), whereas it was 10.0% in the low-apelin group (<70.47 pg/mL) (*p* = 0.004). In multivariate Cox regression analysis, an apelin level <70.47 pg/mL was identified as an independent prognostic factor (HR: 0.16; 95% CI: 0.03–0.94). The cumulative survival rates at days 28, 56, and 180 were 56.1%, 46.7%, and 11.7%, respectively, in the high-apelin group, while these rates were 100%, 100%, and 81.8%, respectively, in the low-apelin group. The mean NIHSS score was significantly higher in the high-apelin group, and a strong, positive, and highly significant correlation was found between serum apelin level and NIHSS score (r = 0.703, *p* < 0.001). **Conclusions:** High serum apelin levels measured within the first 24 h in acute ischemic stroke are associated with increased 28-day and long-term mortality. The association of high endogenous apelin levels with poor prognosis in the clinical setting suggests that apelin is mobilized as a stress response during ischemia, and that elevated circulating levels may actually reflect the severity of the ischemic insult.

## 1. Introduction

Apelin is the endogenous ligand of the G protein–coupled receptor APJ and is widely distributed in many tissues, particularly in the nervous system. Apelin exerts neuroprotective effects against oxidative stress. This pathway protects cells from damage by enhancing the production of antioxidant enzymes [1,2]. Furthermore, apelin subtypes (apelin-36, apelin-12, and apelin-13) promote neuronal survival by suppressing apoptosis and autophagy through the PI3K/Akt, ERK, and mTOR pathways [3,4,5,6,7]. Regarding cerebral edema, apelin alleviates edema by increasing aquaporin-4 (AQP4) expression and preserves the blood–brain barrier. It also supports barrier integrity by inhibiting occludin, claudin-5, endothelin B receptors (ETBR), and matrix metalloproteinases (MMPs) [6,7,8,9,10,11,12,13,14,15,16,17,18,19]. On the inflammatory front, apelin reduces chemokines such as MCP-1 and MIP-1, increases anti-inflammatory cytokines such as IL-10, and protects neural cells by suppressing MMP-9. In the long term, it may promote angiogenesis and restoration of blood flow by elevating VEGF and MMP-9 levels [10,11]. In summary, the apelin/APJ system exerts a neuroprotective effect by regulating the core pathogenic mechanisms of ischemic stroke—namely oxidative stress, apoptosis, cerebral edema, and inflammation—through various signaling pathways. However, further studies are needed to elucidate the exact mechanisms.

Cerebrovascular diseases are mostly caused by brain injuries, and pharmacological interventions have largely focused on post-surgical recovery [12]. The palliative (ameliorative) effect of the apelin/APJ system in brain injuries such as subarachnoid hemorrhage and ischemic stroke has been widely reported, and it has been suggested that the apelin/APJ system may represent a potential therapeutic target for cerebrovascular diseases [13]. Nevertheless, the extent to which these multifaceted neuroprotective effects demonstrated in experimental models translate into clinical prognosis has not been sufficiently investigated. In particular, data regarding whether circulating apelin levels in the acute phase (first 24 h) of ischemic stroke can predict long-term functional recovery remain limited. However, the relative ease with which apelin crosses the blood–brain barrier and its stable measurability in peripheral blood make it an attractive biomarker candidate. Given that imaging modalities, which are considered the gold standard for prognosis determination, are not applicable to every patient [14], a readily accessible, non-invasive, and cost-effective biomarker such as apelin could provide clinicians with valuable guidance. Given that different apelin isoforms exhibit distinct biological activities and variable circulating half-lives, the present study measured total serum apelin levels to provide an integrated assessment of the overall apelinergic response in the acute phase of ischemic stroke. Although isoform-specific measurements would offer more detailed mechanistic insights, total apelin quantification serves as a pragmatic and clinically feasible approach for initial prognostic evaluation, particularly in the emergency setting where rapid biomarker assessment is essential. In the current literature, apelin level measurement after ischemic stroke is not regarded as a routine clinical practice; the published human studies are largely clustered as research-oriented, single-center, or limited multicenter biomarker investigations. A notable feature of these studies is their predominant concentration in East Asia. For instance, Wang et al. [15] investigated the prognostic value of serum apelin-13 levels in a cohort of 244 patients with acute ischemic stroke, Jiang et al. [16] examined the relationship between plasma apelin-17 and apelin-36 levels and collateral circulation, while Yu et al. [17] presented a case–control series comparing MCA occlusion with Moyamoya disease in a Chinese population. These studies clearly demonstrate the research density in East Asia. Güler et al. [18] from Turkey measured serum and urinary apelin levels in acute stroke, and Lee et al. [19] from Republic of Korea monitored blood apelin levels before and after rehabilitation, offering regional but more sporadic contributions. Bohm et al. [20] from Central Europe, on the other hand, did not directly assess post-stroke apelin levels but evaluated plasma apelin-12 measurement in the context of atrial fibrillation screening among patients at high risk of stroke. This pattern suggests that apelin measurement largely accompanies selected research questions and remains predominantly incidental or research-driven in its use. Although the publication timeline extending from 2018 to 2026 indicates sustained interest in the topic, the available studies do not prove that testing frequency has genuinely increased in any particular region. The authors’ calls for large-scale multicenter validation studies [15,16,17,18,19,20,21] underscore that the field is still in its early stages and has yet to be integrated into standardized clinical practice. The aim of this study was to investigate the prognostic role of apelin levels measured within the first 24 h in patients with ischemic stroke.

## 2. Materials and Methods

### 2.1. Study Design

This prospective cohort study was conducted between 2023 and 2025 at Şanlıurfa Suruç State Hospital on patients hospitalized with a diagnosis of acute ischemic stroke. The study protocol was approved by the Ethics Committee of Harran University (approval date: 27 March 2023). All procedures were performed in accordance with the principles of the Declaration of Helsinki. Written informed consent was obtained from all patients included in the study or from their first-degree relatives in cases where patients were unable to provide consent due to impaired consciousness or aphasia.

### 2.2. Sample Size

Sample size was calculated using G*Power 3.1.9.4 (test family: *t*-tests, difference between two independent group means). Assuming an effect size of Cohen’s d = 0.8, α = 0.05, power = 0.80, and a group ratio of 1:1, a total of 42 patients were determined to be required. The study included 44 patients aged between 40 and 82 years who were hospitalized with a diagnosis of acute ischemic stroke due to large artery atherosclerosis. The diagnosis of ischemic stroke was confirmed by clinical findings and neuroimaging methods [cranial computed tomography (CT) and/or diffusion-weighted magnetic resonance imaging (MRI)]. Stroke subtype was determined according to the TOAST (Trial of Org 10172 in Acute Stroke Treatment) classification, and only patients in the large artery atherosclerosis subgroup were included in the study.

**Inclusion Criteria**

- First acute ischemic stroke due to large artery atherosclerosis, confirmed by neuroimaging
- Admission to the hospital within the first 24 h after symptom onset
- Availability of a serum sample obtained within the first 24 h

**Exclusion Criteria**

- Lacunar infarction
- Cardioembolic stroke (valvular heart disease)
- Stroke due to other determined etiologies (e.g., vasculitis, arterial dissection, hypercoagulable states)
- Transient ischemic attack (TIA)
- Previous history of cerebrovascular disease
- Presence of malignancy, advanced-stage liver or renal failure, or systemic inflammatory disease
- Patients who received thrombolytic therapy or endovascular thrombectomy
- Patients with incomplete clinical or laboratory data

### 2.3. Collection and Assessment of Clinical Data

Demographic characteristics, vascular risk factors, and medical histories of all patients were recorded at the time of admission. The following comorbid diseases were documented according to standard diagnostic criteria or based on whether the patients were using the relevant medications. All patients underwent a detailed neurological examination performed by a neurologist. Stroke severity at admission was assessed using the National Institutes of Health Stroke Scale (NIHSS). Functional status of the patients was determined using the modified Rankin Scale (mRS) at discharge and during follow-up.

#### 2.3.1. NIH Stroke Scale (NIHSS)

The National Institutes of Health Stroke Scale (NIHSS) is a standardized measurement tool used to systematically and quantitatively evaluate the severity of neurological deficit in patients with acute ischemic stroke. The NIHSS has high reliability in assessing neurological damage in acute stroke. Inter-observer agreement (ICC = 0.95) and test–retest reliability (ICC = 0.93) are at an excellent level. The scale correlates with infarct volume and is a valid tool for predicting short- and long-term prognosis after stroke.

The NIHSS is a scoring system with proven effectiveness in determining the severity of damage in acute stroke and can be easily administered at the bedside by a physician. The scale evaluates 15 items across 11 categories, including level of consciousness, language function, neglect, visual field loss, extraocular movements, motor strength, ataxia, dysarthria, and sensory loss. The NIHSS is calculated as a total score ranging from 0 to 42. Each category is scored between 0–3 or 0–4, with higher scores indicating more severe neurological deficits. The total NIHSS score is used to classify stroke severity and predict prognosis.
**Total Score****Stroke Severity****Clinical Interpretation**0No symptomsNormal neurological examination1–5Mild strokeApproximately 80% of patients can return home after discharge6–14Moderate strokeGenerally requires acute inpatient rehabilitation>14Severe strokeLong-term care is frequently needed

#### 2.3.2. Modified Rankin Scale (mRS)

The modified Rankin Scale (mRS) is a widely accepted and frequently preferred measurement tool in clinical research, used to assess the level of functional dependence and limitations in activities of daily living in individuals with cerebrovascular disease. The Rankin Scale was first described by Rankin in 1957 and later modified to its current form. The mRS is considered the gold standard for evaluating functional outcomes after stroke and is extensively used as a primary outcome measure in clinical trials comparing the efficacy of acute stroke treatments. The scale evaluates the extent to which the patient requires assistance from others in activities of daily living and is based on a 7-tier scoring system ranging from 0 (no symptoms at all) to 6 (death). In our study, an mRS score of 0–2 was defined as a “good functional outcome,” while a score of 3–6 was defined as a “poor functional outcome.”

### 2.4. Follow-Up and Outcome Measures

All patients were prospectively followed from the day of hospital admission until death or the end of the study period. The primary outcome measures of the study were defined as follows:

- 28-day mortality: Death from any cause within the first 28 days after stroke onset
- Long-term mortality: Death from any cause during the entire follow-up period

All patients were prospectively followed from the day of hospital admission until death or the end of the study period. The maximum follow-up duration was approximately 720 days (24 months) for patients enrolled early in the study period.

Survival status was assessed at pre-specified time points: 28, 56, 84, 112, and 180 days post-stroke. Long-term mortality was defined as death occurring during the entire follow-up period, extending up to 716 days.

Follow-up data were obtained through hospital records, outpatient clinic visits, and structured telephone interviews with patients or their caregivers.

### 2.5. Blood Sample Collection and Biochemical Analysis (Apelin)

Peripheral venous blood samples were obtained from all patients within the first 24 h after symptom onset, following a 12-h fasting period. A single time-point measurement at admission was preferred to establish a standardized and clinically feasible sampling protocol that could be easily implemented in the emergency setting. Blood samples were collected into plain vacuum tubes (BD Vacutainer®, Becton, Dickinson and Company, Franklin Lakes, NJ, USA) without anticoagulant and centrifuged at 4000 rpm for 10 min at +4 °C. The obtained serum samples were divided into equal aliquots (to prevent repeated freeze–thaw cycles) and stored at −80 °C until analysis. Serum samples were transported to the Biochemistry Laboratory of Kahramanmaraş Sütçü İmam University Faculty of Medicine under appropriate cold chain conditions (on dry ice). The ELISA kit used in this study measures total apelin levels (recognizing multiple isoforms including apelin-12, -13, -17, and -36) and does not distinguish between individual isoforms. This approach was preferred to capture the cumulative apelinergic response, as the relative contribution of each isoform to circulating apelin pool in acute ischemic stroke has not been well characterized. Optical density measurements were performed using an automated ELISA microplate reader (BioTek 800™ TS, Winooski, VT, USA), and data were analyzed using Gen5 Version 3.11.19 software. The intra-assay coefficient of variation (CV) was <8%, and the inter-assay CV was <10%. All measurements were performed in duplicate, and results were expressed in pg/mL.

### 2.6. Statistical Analysis

Statistical analyses were performed using Jamovi software (version 2.6.44). Confidence intervals were calculated at the 95% level. Categorical variables were summarized as numbers (*n*) and percentages (%). Normality of continuous variables was assessed using the Shapiro–Wilk test. Continuous variables not following a normal distribution were presented as median (25th–75th percentiles), while those following a normal distribution were presented as mean ± standard deviation (SD). Comparisons of categorical variables between the two groups formed according to the apelin cut-off value (≥70.47 and <70.47) were performed using the Pearson chi-square test. Fisher’s exact test was preferred when the expected frequency was less than 5. For group comparisons of continuous variables, the Mann–Whitney U test (for non-normally distributed variables) or the independent samples *t*-test (for normally distributed variables) was applied. The diagnostic performance of serum apelin levels in predicting mortality was evaluated using Receiver Operating Characteristic (ROC) analysis. The area under the curve (AUC) was calculated and presented with 95% confidence interval. The optimal cut-off value was determined using the Youden index (J = sensitivity + specificity − 1). Based on this cut-off value, sensitivity, specificity, positive and negative likelihood ratios (LR+ and LR−), positive and negative predictive values (PPV and NPV), and overall accuracy were calculated. In survival analysis, the primary endpoint was defined as 28-day mortality. Follow-up time was calculated in days. Kaplan–Meier method was used to generate survival curves for the groups. Differences in survival distributions between groups were compared using the log-rank test. Median survival times and cumulative survival probabilities at specific time points (28, 56, 84, 112, and 180 days) were calculated with 95% confidence intervals. To identify factors affecting mortality, Kaplan–Meier survival analysis and the Cox proportional hazards regression model were used. The overall performance of the model was evaluated using the concordance index (C-index) and the likelihood ratio test. Additionally, the Nagelkerke (Cragg–Uhler) R^2^ value was calculated and presented along with the maximum possible R^2^. A *p*-value < 0.05 was considered statistically significant.

## 3. Results

The mean age of the patients included in the study was 72.2 ± 7.9 years (min: 40–max: 82). During the follow-up period, 29 patients (67.4%) survived, while 14 patients (32.6%) died. During a total person-time of 6688 units, 12 events were recorded, yielding an overall incidence rate of 0.180 (95% CI: 0.090–0.310). Of the 14 initially reported deaths, two were lost to follow-up and therefore not included in the event analysis. The highest event rate was observed in the first 28 days (6 events; rate: 0.860, 95% CI: 0.320–1.880), followed by a sharp decline during days 28–56 (rate: 0.210, 95% CI: 0.010–1.150) and a secondary rise between days 56 and 112 (rates: 0.750 and 0.680, respectively). No events were recorded after day 112 (rates: 0.000 for both later intervals), and the declining person-time in these periods reflects censoring due to loss to follow-up.

**Figure 1:**
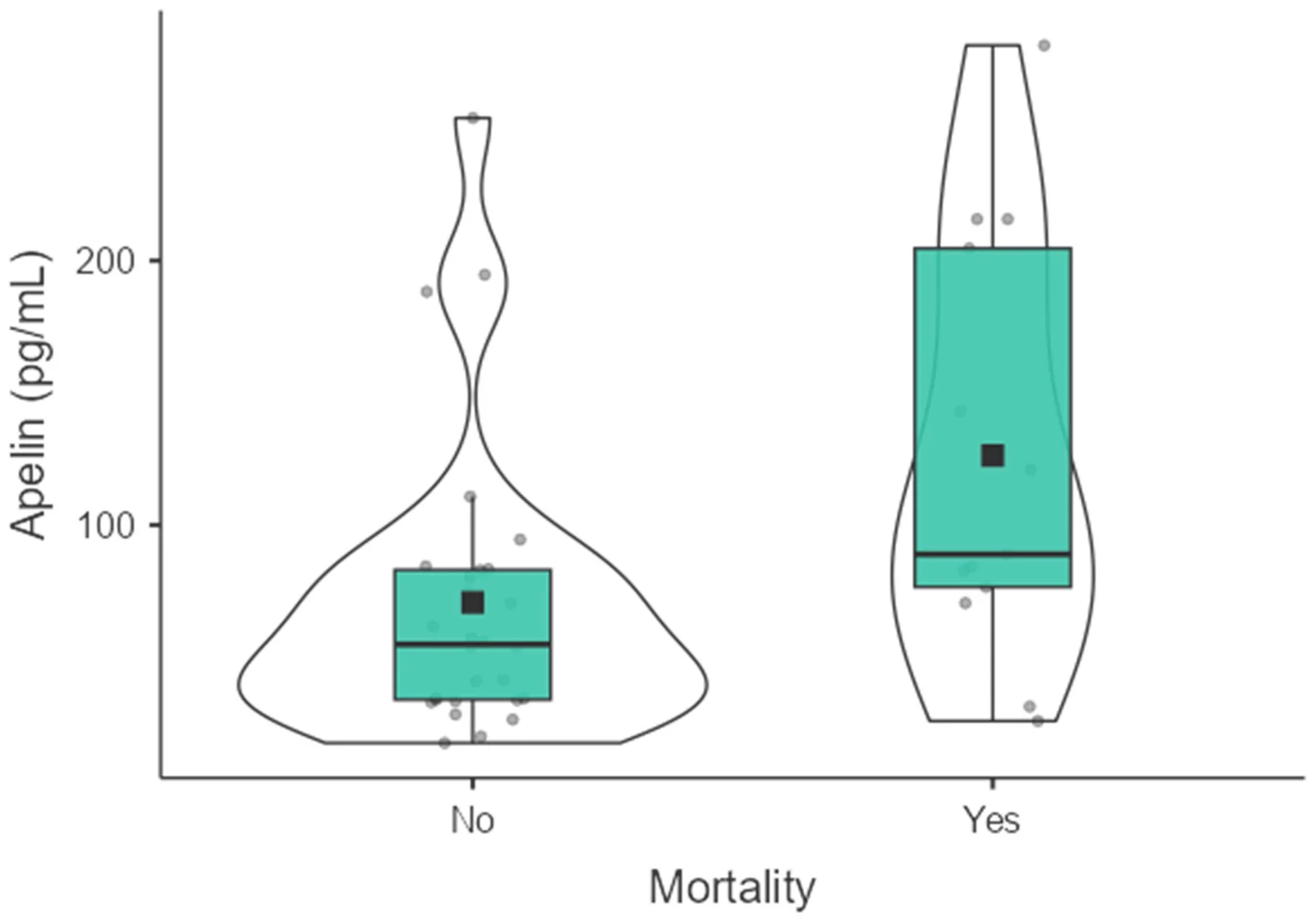
First 24-h apelin levels according to mortality status.

In the comparison between survivors and non-survivors, a statistically significant difference was found in terms of mean age; the mean age of the survivor group was 68.8 ± 7.31 years, whereas that of the deceased group was 79.1 ± 3.23 years (*p* < 0.001). When apelin levels were evaluated as median (IQR), they were determined to be 55.8 pg/mL (34.1–83.4) in the survivor group and 105.0 pg/mL (78.1–213.0) in the deceased group, indicating that apelin levels were significantly higher in the deceased group (*p* = 0.017) (Figure 1). When the distribution of medications prescribed at discharge was examined, the most frequently used combination, Clopidogrel + Acetylsalicylic acid, was observed in 79.3% (*n* = 23) of survivors and 42.9% (*n* = 6) of non-survivors, while warfarin use was observed at a higher rate in the deceased group (28.6%, *n* = 4), and the difference between the groups was significant (*p* = 0.029). No statistically significant differences were found between the groups in terms of sex, occluded artery distribution, stroke localization, etiology, LDL level, number of comorbidities, or number of subsequent cerebrovascular events after apelin measurement (Table 1).

**Figure 2:**
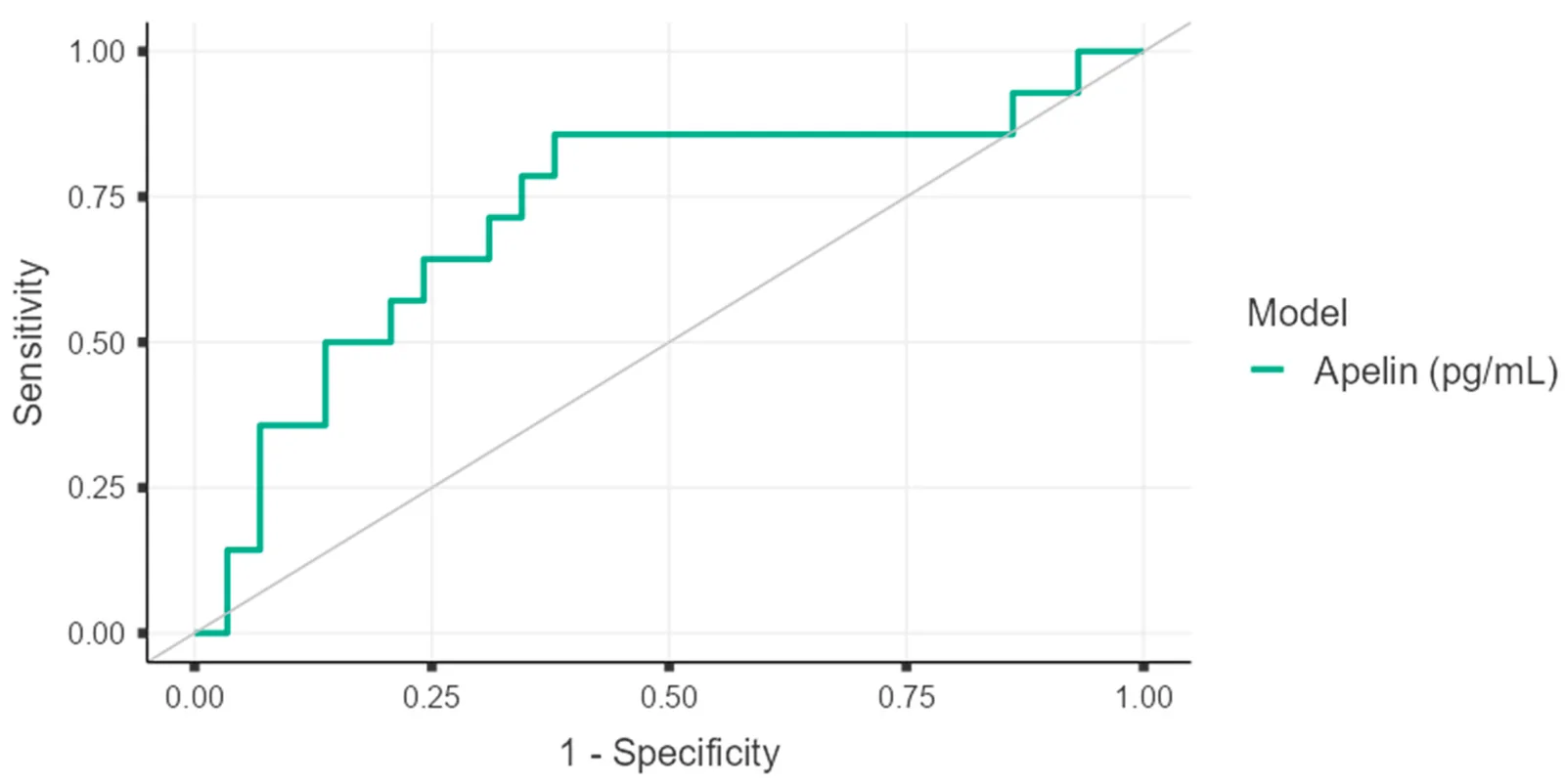
ROC curve of apelin for predicting mortality.

The diagnostic accuracy of serum apelin levels at admission in predicting mortality in patients with ischemic cerebrovascular events was evaluated using ROC analysis. The area under the curve (AUC) for apelin in classifying mortality was calculated as 0.727 (Figure 2). This value indicates that apelin has a statistically significant but moderate discriminative power. The optimal cut-off value determined using the Youden index was ≥70.47. According to this cut-off value, the sensitivity and specificity of apelin levels in classifying mortality were 85.71% and 62.07%, respectively. Overall accuracy was 69.77% (Table 2).

Patients were compared according to their sociodemographic and clinical characteristics based on apelin levels. In the high-apelin group (≥70.47), the mean age was found to be significantly higher compared to the low-apelin group (median 77.0 vs. 68.0; *p* = 0.024). No statistically significant differences were found between the two groups in terms of sex distribution, occluded artery localization, etiology, number of CVD events, LDL levels, or presence of comorbid diseases (*p* > 0.05). The 28-day mortality rate was 52.2% in the high-apelin group, whereas it was 10.0% in the low-apelin group (*p* = 0.004). The mean NIHSS score was found to be significantly higher in the high-apelin group (Table 3).

**Figure 3:**
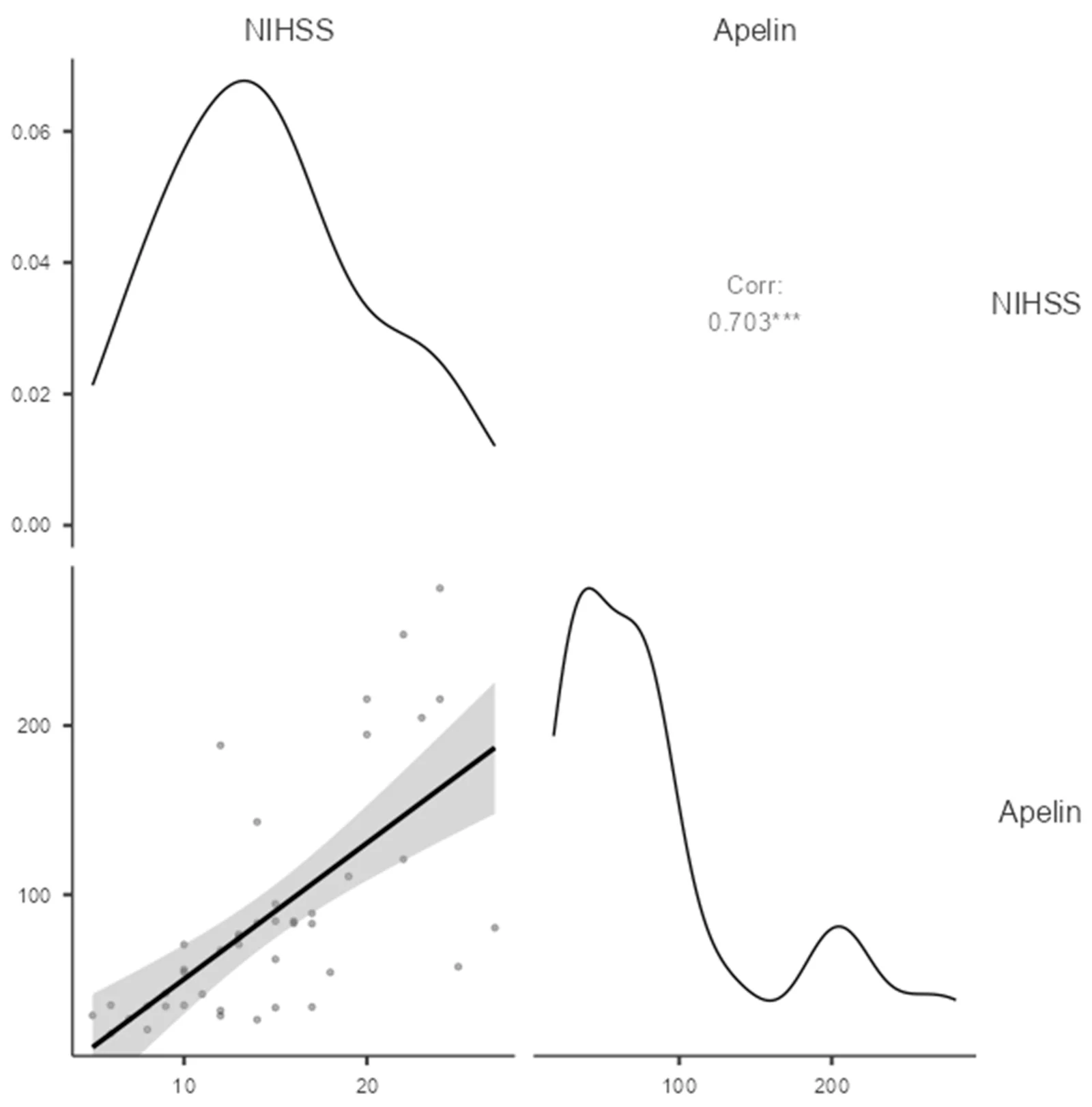
Correlation between apelin and NIHSS. (Note: *** *p* < 0.001)

The relationship between the NIHSS (National Institutes of Health Stroke Scale) score, used to assess stroke severity at admission, and serum apelin levels measured within the first 24 h was evaluated using Spearman’s correlation analysis. The analysis revealed a strong, positive, and highly statistically significant correlation between the NIHSS score and serum apelin levels (Spearman’s rho = 0.703, df = 41, *p* < 0.001) (Figure 3).

**Figure 4:**
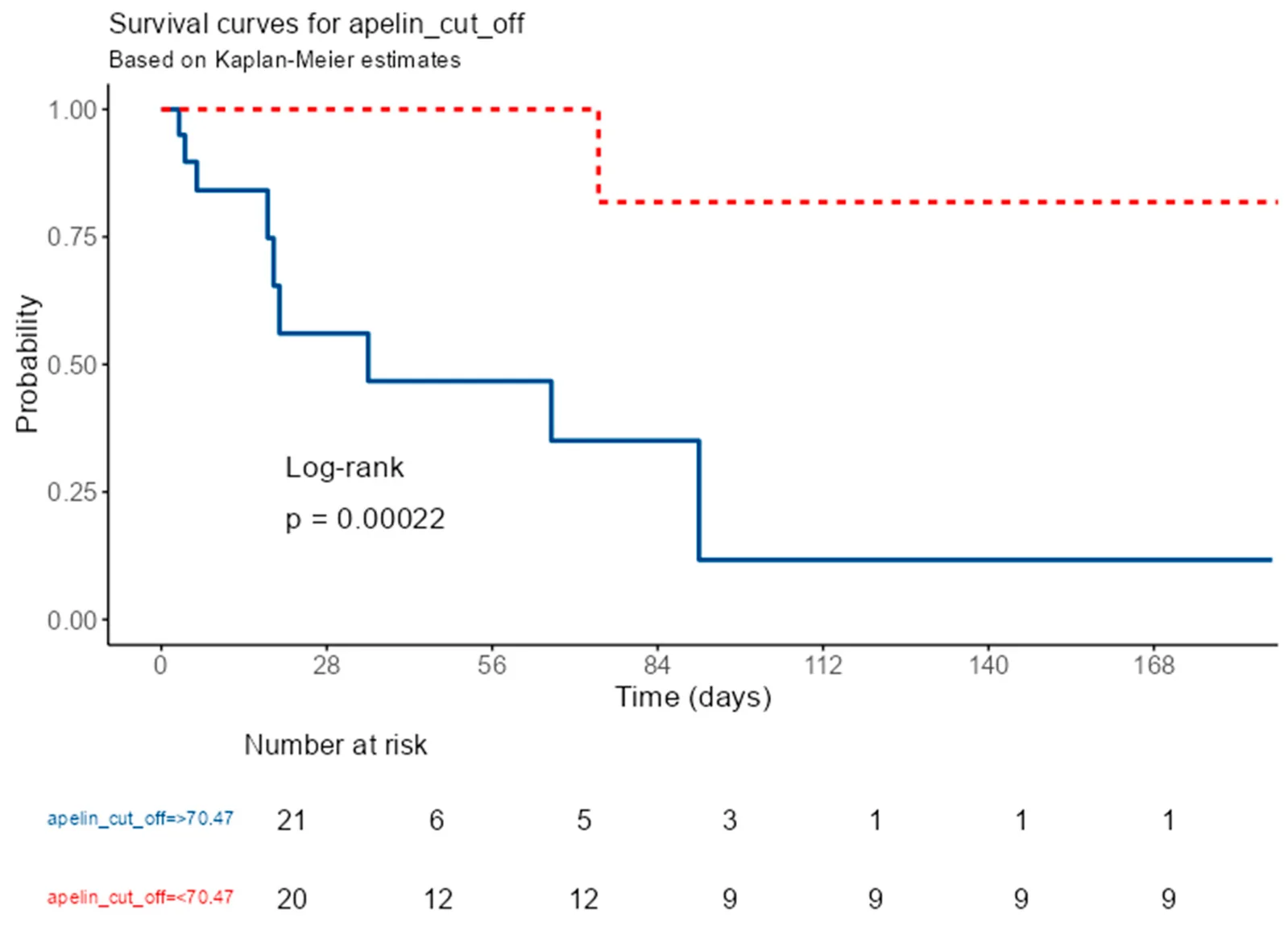
Survival Plot—Apelin cut-off.

The concordance index of the Cox model examining the effect of apelin levels on survival was 0.769 (SE = 0.038), indicating that the model has good discriminative power. The likelihood ratio test statistic (χ^2^ = 13.150, df = 1, *p* < 0.001) confirmed that the model was statistically significant. Significantly longer survival was observed in the low-apelin group (<70.47) compared to the high-apelin group (≥70.47) (log-rank test equivalent: *p* < 0.001). In the high-apelin group, the median survival was calculated as 35.0 days (95% CI: 19.0–NA), whereas the median survival could not be reached in the low-apelin group during the follow-up period (the survival curve did not fall below the 50% level). In univariate Cox proportional hazards regression analysis, the hazard ratio (HR) for the low-apelin group (<70.47) compared to the high-apelin group was 0.09 (*p* = 0.002). In the high-apelin group, the survival probability at day 28 was 56.1%, at day 56 was 46.7%, at day 84 was 35.0%, and at day 112 and beyond was 11.7%. In the low-apelin group, survival at days 28 and 56 was 100%, at day 84 was 81.8%, and at days 112 and 180 remained at 81.8% (Figure 4, Table 4).

To evaluate the independent prognostic value of serum apelin, multivariable Cox proportional hazards regression models were constructed adjusting for age, a well-established predictor of mortality in stroke patients. The multivariable model demonstrated good discriminative ability with a concordance index of 0.781 (SE = 0.054). The likelihood ratio test confirmed the overall significance of the model (χ^2^ = 14.293, df = 2, *p* = 0.001), with the model explaining 29.4% of the variance in mortality risk (R-squared = 0.294). As presented in Table 5, univariate Cox regression analysis demonstrated that serum apelin level below the cut-off value of 70.47 pg/mL was significantly associated with reduced all-cause mortality risk (HR: 0.09, 95% CI: 0.02–0.43, *p* = 0.002). Age was also significantly associated with mortality in univariate analysis (HR: 1.14, 95% CI: 1.03–1.27, *p* = 0.010). After adjusting for age in the multivariable model, low serum apelin remained an independent protective factor against all-cause mortality (adjusted HR: 0.16, 95% CI: 0.03–0.95, *p* = 0.044). Notably, the prognostic significance of age was attenuated in the multivariable model (adjusted HR: 1.07, 95% CI: 0.94–1.21, *p* = 0.297), suggesting that serum apelin provides prognostic information independent of age.

**Figure 5:**
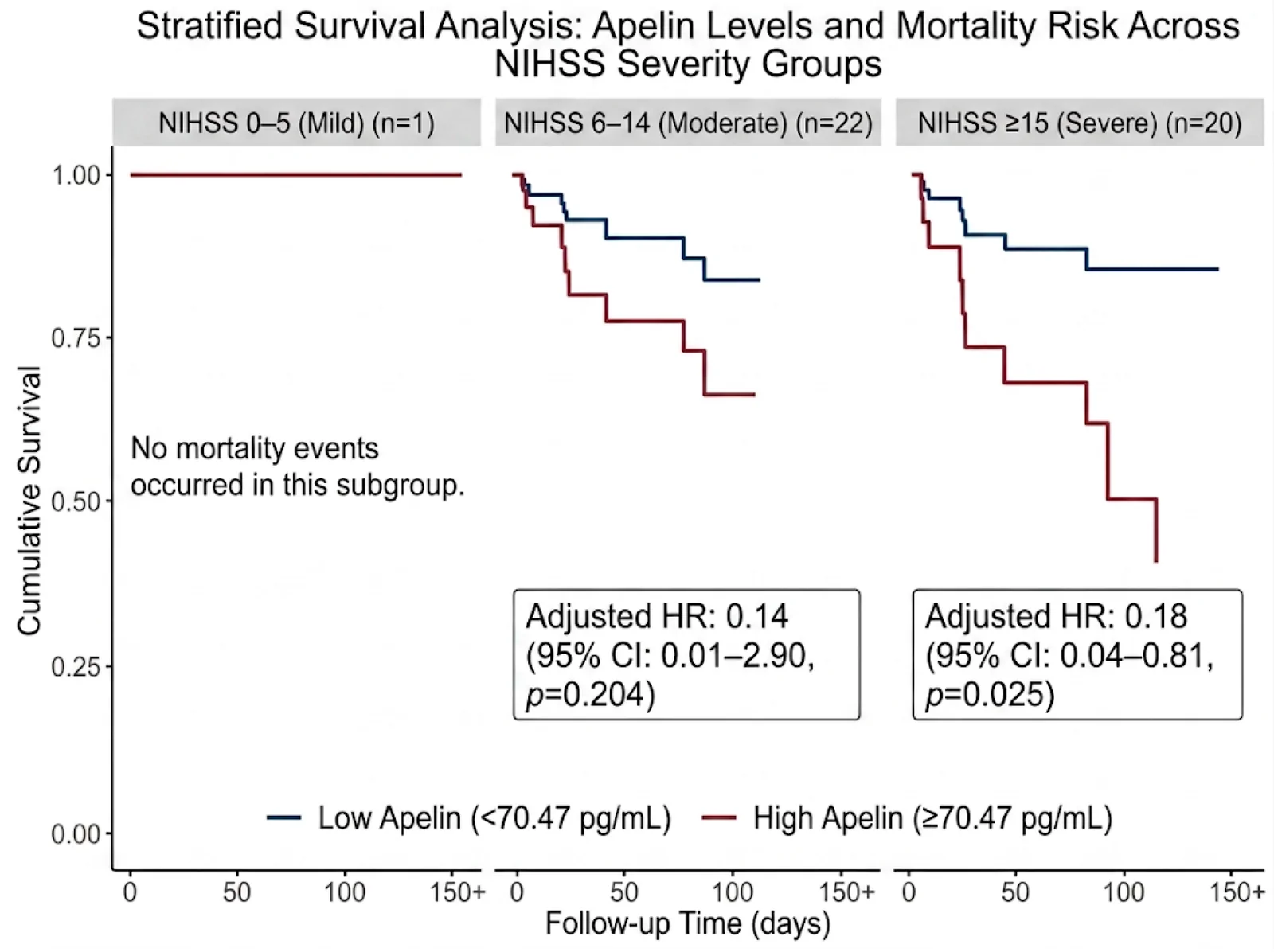
Adjusted survival curves showing the association between low versus high serum apelin levels and mortality risk, stratified by NIHSS severity subgroups (mild: 0–5, moderate: 6–14, severe: ≥15). Hazard ratios (HR) and 95% confidence intervals (CI) are presented for each subgroup.

To further validate the independent prognostic value of apelin across different levels of stroke severity, we performed stratified survival analyses according to NIHSS subgroups using established cut-off values (mild: 0–5, moderate: 6–14, severe: ≥15). As shown in Figure 5, the protective effect of low apelin was most pronounced in patients with severe stroke (NIHSS ≥ 15; HR: 0.18, 95% CI: 0.04–0.81, *p* = 0.025). In the moderate stroke subgroup, a trend toward reduced mortality was observed, although statistical significance was not reached (HR: 0.14, 95% CI: 0.01–2.90, *p* = 0.204), likely due to the limited number of events. No mortality events occurred in the mild stroke subgroup during follow-up, precluding further analysis. Figure 5 clearly demonstrates that within each NIHSS subgroup, low apelin levels were consistently associated with better survival compared to high apelin levels, particularly in the severe stroke subgroup.

## 4. Discussion

The apelin/APJ system has been associated with multifaceted neuroprotective mechanisms in experimental models, including suppression of oxidative stress, inhibition of apoptosis, reduction in cerebral edema, and modulation of the inflammatory response [1,2,3,4,5,6,7,8,9,10,11]. These mechanisms suggest that apelin is mobilized as an endogenous protective response in damaged brain tissue following cerebral ischemia. However, data regarding how these experimental findings translate into clinical prognosis are limited. Although experimental studies in the literature demonstrate that apelin is neuroprotective in ischemic stroke [1,2,3,4,5,6,7,8,9,10,11], clinical studies have unexpectedly shown conflicting results regarding the association between circulating apelin levels and disease prognosis. The basis of this discrepancy may lie in temporal expression differences in apelin during ischemia and reperfusion phases, possible differences between peripheral circulating apelin levels and local brain apelin levels, and heterogeneity in the biological activities of different apelin molecular forms (apelin-12, -13, -17, -36). In particular, the role of circulating apelin concentrations in the acute phase (first 24 h) in predicting short- and long-term patient survival has not been sufficiently elucidated. In this context, our study aimed to evaluate the relationship between apelin and prognosis during the acute ischemic period. A notable finding of our study is the significant age difference between survivors and non-survivors. Patients who died during follow-up were approximately 10 years older than those who survived (79.1 ± 3.23 years vs. 68.8 ± 7.31 years, *p* < 0.001). This finding is consistent with well-established knowledge that advanced age is one of the strongest predictors of poor outcomes and mortality after ischemic. However, it is important to note that the age difference between the two apelin groups (high vs. low) was also significant (74.7 ± 7.9 years vs. 69.3 ± 7.1 years, *p* = 0.027), suggesting that age may be a confounding factor in the relationship between apelin and mortality. In the multivariate Cox regression analysis, age was not an independent predictor of mortality when apelin was included in the model (HR: 1.07, 95% CI: 0.94–1.21, *p* = 0.297), whereas apelin level below 70.47 pg/mL remained an independent protective factor (HR: 0.16, 95% CI: 0.03–0.95, *p* = 0.044). This indicates that the prognostic value of apelin is independent of age, and that the association between elevated apelin and poor prognosis is not merely a reflection of older age in the high-apelin group. Nevertheless, the significant age difference between outcome groups underscores the importance of considering age as a potential confounder in future studies and highlights the need for age-matched or age-adjusted analyses.

In our study, patients who died during the follow-up period had significantly higher median serum apelin levels compared to the survivor group. ROC analysis revealed that apelin levels had moderate but statistically significant discriminative power in predicting mortality (AUC: 0.727; 95% CI: 0.550–0.903), and the optimal cut-off value was determined as ≥70.47 pg/mL. Survival analyses based on this cut-off value demonstrated that the 28-day mortality rate was 52.2% in the high-apelin group, whereas it was only 10.0% in the low-apelin group (*p* = 0.004). Kaplan–Meier curves revealed that survival probability in the high-apelin group declined to 11.7% by day 180, whereas it remained at 81.8% in the low-apelin group. Furthermore, the significantly higher mean NIHSS score in the high-apelin group and the strong, positive correlation between serum apelin levels and NIHSS score (r = 0.703, *p* < 0.001) strongly support the association of elevated apelin with more severe neurological deficits. In multivariate Cox regression analysis, apelin levels < 70.47 pg/mL emerged as an independent prognostic factor associated with lower mortality risk, independent of age (HR: 0.16; 95% CI: 0.03–0.94; *p* = 0.043).

In the study by Khaksari et al., the effects of the peptide molecule apelin-13 on brain injury and edema were investigated in a transient focal cerebral ischemia model in rats. Apelin-13 at doses of 50 and 100 μg significantly reduced total brain injury volume by 45% and 55%, respectively. However, no significant effect on brain injury was observed at the lowest dose of 25 μg. Post-ischemic cerebral edema was markedly reduced with 50 and 100 μg apelin-13 administration. Apelin-13 provided protection by reducing caspase-3 activation, which plays a critical role in the cell death process. Notably, the 100 μg dose demonstrated a strong anti-apoptotic effect, reducing the number of caspase-3-positive cells by 69%. One striking finding of the study was that although apelin-13 reduced physical brain injury and edema, it did not significantly improve neurological functional deficits. The study provided the first evidence that apelin-13 may be an effective therapeutic target in a dose-dependent manner for protecting brain tissue and reducing edema in stroke [22].

The experimental findings reported by Khaksari et al. [22] demonstrate dose-dependent protective effects of apelin-13 on brain tissue following cerebral ischemia. However, another noteworthy finding of that study was that despite reducing physical brain injury and edema, apelin-13 failed to significantly improve neurological functional deficits. This suggests that there may not be a direct parallel between apelin’s structural damage-reducing effects and functional recovery. In our study, the finding that high apelin levels were associated with higher NIHSS scores and increased mortality indicates that the neuroprotective effects observed in experimental models may not directly translate to the clinical setting. This paradox may stem from the fundamental difference between preclinical studies, which typically involve exogenous administration of apelin (therapeutic approach), and clinical studies, which measure endogenous circulating apelin levels (prognostic approach).

In the study by Jiang et al., plasma apelin-17 and apelin-36 levels in ischemic stroke patients with good collateral circulation were found to be significantly higher than both patients with poor collateral circulation and healthy individuals. In addition, nitric oxide (NO) levels in these patients were higher compared to healthy participants. In vitro experiments demonstrated that apelin-17 significantly attenuated vasoconstriction in cerebral arteries (rat basilar artery). This effect was determined to be endothelium-dependent. Apelin-17 was found to significantly reduce infarct volume in the brain. It has been suggested that plasma apelin-17 levels may serve as a useful biomarker for predicting disease prognosis and monitoring the effects of clinical treatments in ischemic stroke patients [16].

In the study by Wang et al., serum apelin-13 levels in patients with acute ischemic stroke (38.63 pg/mL) were found to be significantly lower compared to healthy controls (42.50 pg/mL). A negative correlation was found between apelin-13 levels and stroke severity (measured by NIHSS score). Patients with milder stroke (NIHSS score ≤ 3) were found to have higher apelin-13 levels compared to those with more severe stroke. At the 3-month follow-up, high apelin-13 levels were observed to reduce the risk of death or severe disability. Furthermore, adding apelin-13 to existing clinical models was found to improve the predictive accuracy for the patient’s 3-month recovery outcome [15]. In the study by Wu et al., it was reported that during ischemia in ischemic stroke, apelin and APLNR expression increases due to hypoxia and glucose deprivation (this increase is primarily triggered by HIF-1α and SP1 transcription factors), whereas during the reperfusion phase, apelin and APLNR expression decreases. These results demonstrate that the apelinergic system serves as a protective mechanism during ischemic stroke, but its levels fluctuate across different phases of stroke (ischemia vs. reperfusion), which is critical for treatment timing [23].

While Wang et al. [15] reported that serum apelin-13 levels in acute ischemic stroke were lower than in healthy controls and that high apelin-13 levels were associated with better functional recovery, our study found that high apelin levels were associated with increased mortality and worse neurological status. This discrepancy may have multiple reasons. First, Wang et al. measured the specific apelin-13 isoform, whereas our study measured total apelin levels; different isoforms such as apelin-13, apelin-17, and apelin-36 may differ in their biological activities and circulating kinetics. Second, differences between study populations (e.g., stroke severity, rates of reperfusion therapy receipt, comorbid conditions) may have contributed to these opposing results. Third, and perhaps most importantly, the time interval in which apelin levels are measured is critical; as highlighted by Wu et al. [23], apelin levels change temporally across ischemia and reperfusion phases. The time window for apelin measurement in our study may differ from that of Wang et al.’s study, which could explain the discordance in results.

The temporal expression model proposed by Wu et al. [23] provides a critical framework for interpreting our findings. While ischemia-induced hypoxia and glucose deprivation increase apelin expression via HIF-1α and SP1, ER stress (ATF4/p38-MAPK pathway) and inflammatory STAT3 signaling suppress apelin production during the reperfusion phase. In our study, apelin levels were assessed during the early phase of ischemia. Therefore, high apelin levels may be interpreted as a “stress response” reflecting the severity of ischemia; that is, elevated apelin is not directly a cause of death but rather a marker of more severe ischemia. This hypothesis is supported by the significantly higher NIHSS scores in the high-apelin group in our study. Alternatively, elevated apelin in some patients may indicate insufficient reperfusion or ongoing ischemia. In this case, high apelin levels may predict poor prognosis as a marker of failed reperfusion or persistent ischemic stress. However, because our study only includes a single measurement at the early ischemic phase, we are unable to capture the subsequent decline in apelin levels that would be expected during reperfusion. This limitation prevents us from determining whether patients with high apelin levels at admission experience a gradual decrease over time, or whether persistently elevated apelin indicates ongoing ischemia and failed reperfusion. Serial measurements would have allowed us to test this hypothesis directly and to distinguish between apelin as a ‘snapshot’ marker of initial injury severity versus a ‘dynamic’ marker of evolving pathophysiology. Despite this limitation, the strong correlation between apelin levels and NIHSS scores supports the interpretation that elevated apelin reflects the severity of the initial ischemic insult.

Studies have demonstrated that apelin and APLNR expression levels may change in a time-dependent manner during ischemic stroke. In the heart, expression of these two molecules increases only in the early phase following ischemia [24]. Similarly, glucose deprivation initially increases apelin and APLNR levels, but leads to a significant decrease in these levels at later stages [25]. Multiple mechanisms involved in ischemic stroke may underlie this late-phase decrease. ER stress plays an important role in cerebral ischemia/reperfusion injury and is activated only during the reperfusion phase, not during ischemia [26,27,28]. ATF4 in the ER stress pathway suppresses apelin production via a p38-MAPK-dependent mechanism [29], potentially contributing to the decline in apelin during reperfusion; subsequently, APLNR levels regress due to decreased apelin. Similarly, STAT3, which is involved in inflammatory responses, directly regulates the apelin gene promoter [30,31]. Since STAT3, like ER stress, is activated only during the reperfusion phase, and inhibition of this signaling has been shown to significantly improve ischemic stroke outcomes, STAT3 is thought to contribute to the reduction in apelin expression during reperfusion [32].

Although apelin-13 (APN) is a potent neuroprotective molecule, its therapeutic efficacy is limited because it cannot cross the blood–brain barrier (BBB). In the study by Ma et al., a delivery system was developed to transport APN directly to the injured region of the brain. The developed nanoparticles were proven to effectively cross the BBB and selectively accumulate in ischemic-inflammatory regions of the brain in both in vitro and in vivo experiments. In mouse models, these nanoparticles were observed to significantly reduce infarct volume, improve neurological scores, alleviate cerebral edema, and preserve cognitive abilities [33]. These findings highlight that the route of administration is a critical determinant of apelin’s therapeutic efficacy. While direct brain delivery or nanoparticle-based approaches show promise for harnessing apelin’s neuroprotective properties, peripheral endogenous apelin appears to function primarily as a marker of systemic stress rather than as an effective central nervous system therapeutic agent. This distinction between exogenous therapeutic apelin and endogenous circulating apelin is essential for understanding the paradoxical relationship observed in our study, where high apelin levels were associated with poor prognosis rather than neuroprotection.

In the study by Peng et al., apelin-13 treatment in ischemic stroke was found to significantly improve post-stroke neurological functions; it reduced infarct volume, cerebral edema, and tissue loss. Apelin-13 preserved blood–brain barrier integrity by reducing vascular leakage and albumin extravasation. Additionally, it suppressed neuronal apoptosis by reducing caspase-3 levels. It was emphasized that apelin-13 suppresses neuroinflammation by activating the SIRT1/NF-κB axis and is a promising therapeutic candidate for promoting post-stroke recovery [34]. In the study by Li et al., while the viability rate decreased in neural stem cells deprived of oxygen and glucose, administration of 100 nM apelin-13 significantly alleviated this decline, increased cell survival rate, and markedly improved migration capacity [35].

The studies by Ma et al. [33] and Peng et al. [34] have advanced the therapeutic potential of apelin-13 by developing delivery systems that transport its neuroprotective effects directly to brain tissue. However, since our findings demonstrate that high endogenous apelin levels are associated with poor prognosis, they suggest that caution should be exercised in the clinical application of exogenous apelin therapy. For apelin therapy to be beneficial, it must be administered at the right time (e.g., at the onset of reperfusion), at the right dose, and with a formulation capable of crossing the blood–brain barrier. Our study indicates that in clinical trials of apelin therapy, patient selection criteria should be carefully determined, keeping in mind that patients with high basal apelin levels may potentially not benefit from treatment or may even be harmed. It would be more rational to develop personalized treatment strategies based on the patient’s endogenous apelin level and the phase of stroke when targeting the apelin/APLNR axis.

### Limitations

Our study has several limitations. First, our sample size is relatively small, and the findings need to be validated in larger populations. Second, and perhaps most critically, apelin levels were measured at a single time point only at admission (within the first 24 h). Serial measurements at subsequent time points (e.g., 48 h, 72 h, and 7 days post-onset) could have better revealed the prognostic value of temporal changes in apelin and allowed us to track the dynamic evolution of the apelinergic response throughout the ischemia–reperfusion continuum. Without longitudinal data, our study cannot determine whether the observed association between high apelin and poor prognosis reflects (i) the severity of the initial ischemic insult, (ii) failure of reperfusion, or (iii) individual variability in the temporal trajectory of apelin response. Future prospective studies with serial apelin measurements covering both ischemic and reperfusion phases are urgently needed to clarify this distinction and to establish the optimal timing for apelin-based prognostic assessment. Third, and most importantly, we measured total apelin levels rather than quantifying individual isoforms (apelin-12, -13, -17, -36) separately. As highlighted in the Introduction, these isoforms exhibit distinct biological activities, receptor binding affinities, and half-lives. Consequently, our study design does not allow us to determine which specific isoform is predominantly responsible for the observed association with mortality, nor whether different isoforms might have opposing prognostic effects. The ELISA kit used in this study recognizes epitopes common to multiple apelin isoforms, and therefore provides an integrated measure of the total apelinergic response. While this approach reflects the cumulative biological signal in the circulation, future studies employing isoform-specific assays are warranted to elucidate the distinct prognostic roles of each apelin subtype. Fourth, patients’ receipt of reperfusion therapy (thrombolytic or endovascular treatment) may have influenced the relationship between apelin levels and prognosis. Finally, the study is single-center, and our results need to be supported by multicenter studies.

## 5. Conclusions

The findings of our study demonstrate that high serum apelin levels at admission in acute ischemic stroke are associated with more severe neurological deficits and increased short- and long-term mortality risk. In contrast to the neuroprotective effects observed in experimental models, this finding suggests that endogenous apelin may serve as a stress biomarker reflecting ischemia severity rather than a protective marker in the clinical setting. The temporal expression dynamics of the apelin/APLNR system during ischemia and reperfusion phases are the most likely mechanism underlying this paradoxical relationship. Future prospective studies measuring apelin isoforms separately, incorporating serial measurements (covering both ischemia and reperfusion phases), and including larger patient populations are needed.

## Figures and Tables

**Table 1:** Comparison of demographic, clinical, and laboratory characteristics of acute ischemic stroke patients included in the study according to mortality status.

Variables	Survivors (*n* = 29)	Non-Survivors (*n* = 14)	Total (*n* = 43)	*p*
Age (years), Mean ± S.D.	68.8 ± 7.31	79.1 ± 3.23	72.2 ± 7.9	<0.001
Sex, *n* (%)				0.526
Female	13 (44.8)	8 (57.1)	21 (48.8)	
Male	16 (55.2)	6 (42.9)	22 (51.2)	
Occluded Artery, *n* (%)				0.791
MCA	7 (24.1)	6 (42.9)	13 (30.2)	
ACA	2 (6.9)	0 (0.0)	2 (4.7)	
Lenticulostriate	11 (37.9)	5 (35.7)	16 (37.2)	
PCA	3 (10.3)	1 (7.1)	4 (9.3)	
PICA	6 (20.7)	2 (14.3)	8 (18.6)	
Stroke Location, *n* (%)				0.720
Anterior circulation	20 (69.0)	11 (78.6)	31 (72.1)	
Posterior circulation	9 (31.0)	3 (21.4)	12 (27.9)	
Apelin (pg/mL), Median (IQR)	55.8 (34.1–83.4)	105.0 (78.1–213.0)	74.9 (34.6–115.9)	0.017
Etiology, *n* (%)				0.614
ICA stenosis	4 (13.8)	4 (28.6)	8 (18.6)	
Atrial fibrillation	6 (20.7)	4 (28.6)	10 (23.3)	
Atherosclerosis	13 (44.8)	6 (42.9)	19 (44.2)	
Other	4 (13.8)	0 (0.0)	4 (9.3)	
ICA + AF	1 (3.4)	0 (0.0)	1 (2.3)	
ICA + Atherosclerosis	1 (3.4)	0 (0.0)	1 (2.3)	
LDL (mg/dL), Median (IQR)	107.0 (92.0–130.0)	114.0 (109.0–128.5)	110.5 (95.0–130.5)	0.303
Number of Comorbidities, *n* (%)				1.000
1 comorbidity	25 (86.2)	13 (92.9)	38 (88.4)	
2 comorbidities	4 (13.8)	1 (7.1)	5 (11.6)	
Cerebrovascular Events After Apelin, Median (IQR)	4.0 (1.0–4.0)	3.5 (2.0–4.0)	4.0 (1.0–4.0)	0.534
Discharge Medication, *n* (%)				0.029
Clopidogrel	0 (0.0)	1 (7.1)	1 (2.3)	
NOAC	1 (3.4)	1 (7.1)	2 (4.7)	
Warfarin	3 (10.3)	4 (28.6)	7 (16.3)	
In-hospital death	0 (0.0)	1 (7.1)	1 (2.3)	
Clopidogrel + Acetylsalicylic acid	23 (79.3)	6 (42.9)	29 (67.4)	
ASA + NOAC	0 (0.0)	1 (7.1)	1 (2.3)	
Warfarin + Clopidogrel	2 (6.9)	0 (0.0)	2 (4.7)	

Note: Follow-up data could not be obtained for one patient; this patient was excluded from the analysis, and statistical evaluations were performed on 43 patients.

**Table 2:** Performance of apelin levels in predicting 28-day mortality.

Metric/Variable	Value	95% CI (Lower)	95% CI (Upper)
AUC (Apelin)	0.727	0.550	0.903
Optimal Cut-off Value	≥70.4745	–	–
Sensitivity	85.71%	57.19%	98.22%
Specificity	62.07%	42.26%	79.31%
Positive Likelihood Ratio (LR+)	2.260	1.354	3.772
Negative Likelihood Ratio (LR−)	0.230	0.062	0.857
Positive Predictive Value (PPV)	52.17%	39.52%	64.55%
Negative Predictive Value (NPV)	90.00%	70.74%	97.10%
Accuracy	69.77%	53.87%	82.82%
Prevalence	32.56%	19.08%	48.54%

**Table 3:** Comparison of demographic, clinical, and laboratory characteristics of patients according to apelin cut-off value.

Variable	Apelin ≥ 70.47 pg/mL (*n* = 23)	Apelin < 70.47 pg/mL (*n* = 20)	Total (*n* = 43)	*p*
Age (years), Mean (SD)	74.7 (7.9)	69.3 (7.1)	72.2 (7.9)	0.027
Sex, *n* (%)				0.763
Female	12 (52.2)	9 (45.0)	21 (48.8)	
Male	11 (47.8)	11 (55.0)	22 (51.2)	
Occluded Artery, *n* (%)				0.967
MCA	8 (34.8)	5 (25.0)	13 (30.2)	
ACA	1 (4.3)	1 (5.0)	2 (4.7)	
Lenticulostriate	8 (34.8)	8 (40.0)	16 (37.2)	
PCA	2 (8.7)	2 (10.0)	4 (9.3)	
PICA	4 (17.4)	4 (20.0)	8 (18.6)	
Stroke Location, *n* (%)				1.000
Anterior circulation	17 (73.9)	14 (70.0)	31 (72.1)	
Posterior circulation	6 (26.1)	6 (30.0)	12 (27.9)	
Etiology, *n* (%)				0.587
ICA stenosis	5 (21.7)	3 (15.0)	8 (18.6)	
Atrial fibrillation	4 (17.4)	6 (30.0)	10 (23.3)	
Atherosclerosis	12 (52.2)	7 (35.0)	19 (44.2)	
Other	2 (8.7)	2 (10.0)	4 (9.3)	
ICA + AF	0 (0.0)	1 (5.0)	1 (2.3)	
ICA + Atherosclerosis	0 (0.0)	1 (5.0)	1 (2.3)	
LDL (mg/dL), Mean (SD)	114.2 (36.7)	115.0 (35.2)	114.6 (35.5)	0.937
Cerebrovascular Events After Apelin, Mean (SD)	3.0 (1.4)	3.0 (1.4)	3.0 (1.4)	0.826
Discharge Medication, *n* (%)				0.666
Clopidogrel	1 (4.3)	0 (0.0)	1 (2.3)	
NOAC	1 (4.3)	1 (5.0)	2 (4.7)	
Warfarin	4 (17.4)	3 (15.0)	7 (16.3)	
In-hospital death	1 (4.3)	0 (0.0)	1 (2.3)	
Clopidogrel + Acetylsalicylic acid	16 (69.6)	13 (65.0)	29 (67.4)	
ASA + NOAC	0 (0.0)	1 (5.0)	1 (2.3)	
Warfarin + Clopidogrel	0 (0.0)	2 (10.0)	2 (4.7)	
Mortality, *n* (%)				0.004
Survivor	11 (47.8)	18 (90.0)	29 (67.4)	
Non-survivor	12 (52.2)	2 (10.0)	14 (32.6)	
mRS				0.147
Bed-dependent	6 (54.5)	1 (12.5)	7 (36.8)	
Independent	5 (45.5)	7 (87.5)	12 (63.2)	
NIHSS, Mean ± SD	17.4 ± 4.54	11.3 ± 4.89	14.5 ± 5.59	<0.001

**Table 4:** Survival analysis according to apelin cut-off value.

Parameter	Apelin ≥ 70.47	Apelin < 70.47
Number of events (mortality)	10/21 (47.6%)	2/20 (10.0%)
Median survival (days)	35.0 (95% CI: 19.0–NA)	Survival curve did not fall below 50%
Mean survival (rmean, days)	160 ± 107	869 ± 113
Survival at day 28 (%)	56.1 (95% CI: 33.9–92.6)	100.0
Survival at day 56 (%)	46.7 (95% CI: 25.2–86.6)	100.0
Survival at day 84 (%)	35.0 (95% CI: 15.2–80.9)	81.8 (95% CI: 61.9–100.0)
Survival at day 112 (%)	11.7 (95% CI: 1.9–71.1)	81.8 (95% CI: 61.9–100.0)
Survival at day 180 (%)	11.7 (95% CI: 1.9–71.1)	81.8 (95% CI: 61.9–100.0)
HR	Reference	0.09 (95% CI: 0.02–0.43), *p* = 0.002
Cox model concordance	0.769 (SE = 0.038)	–
Likelihood ratio test	χ^2^ = 13.150 (df = 1, *p* = 0.000)	–

**Table 5:** Multivariable Cox Regression Analysis for Predictors of Mortality.

Dependent		All	HR (Univariable)	HR (Multivariable)
Apelin cut-off	>70.47	21 (51.2)	-	-
	<70.47	20 (48.8)	0.09 (0.02–0.43, *p* = 0.002)	0.16 (0.03–0.95, *p* = 0.044)
Age	Mean (SD)	71.9 (7.9)	1.14 (1.03–1.27, *p* = 0.010)	1.07 (0.94–1.21, *p* = 0.297)

Model Performance Metrics: Number of patients = 41, Number of events = 12, Concordance index = 0.781 (SE = 0.054), R-squared = 0.294 (Max possible = 0.824), Likelihood ratio test = 14.293 (df = 2, *p* = 0.001) This model is stratified by: NIHSS severity group. Hazard ratios are not shown for stratification variables as they are used to create separate baseline hazard functions.

## Data Availability

The data presented in this study are available on request from the corresponding author due to (specify the reason for the restriction).

## Abbreviations

ACA	Anterior Cerebral Artery
ACS	American Chemical Society
AF	Atrial Fibrillation
APJ	Apelin Receptor (G protein-coupled receptor)
APN	Apelin-13
AQP4	Aquaporin-4
ASA	Acetylsalicylic Acid
ATF4	Activating Transcription Factor 4
AUC	Area Under the Curve
BBB	Blood–Brain Barrier
CI	Confidence Interval
C-index	Concordance Index
CT	Computed Tomography
CV	Coefficient of Variation
CVD	Cerebrovascular Disease
Df	Degrees of Freedom
ELISA	Enzyme-Linked Immunosorbent Assay
ER	Endoplasmic Reticulum
ETBR	Endothelin B Receptor
HIF-1α	Hypoxia-Inducible Factor 1-Alpha
HR	Hazard Ratio
ICC	Intraclass Correlation Coefficient
ICA	Internal Carotid Artery
IQR	Interquartile Range
LDL	Low-Density Lipoprotein
LR+	Positive Likelihood Ratio
LR−	Negative Likelihood Ratio
MCA	Middle Cerebral Artery
MMP	Matrix Metalloproteinase
MRI	Magnetic Resonance Imaging
mRS	modified Rankin Scale
NF-κB	Nuclear Factor Kappa-B
NIHSS	National Institutes of Health Stroke Scale
NO	Nitric Oxide
NOAC	Non-Vitamin K Antagonist Oral Anticoagulant
NPV	Negative Predictive Value
PCA	Posterior Cerebral Artery
PICA	Posterior Inferior Cerebellar Artery
PI3K/Akt	Phosphoinositide 3-Kinase/Protein Kinase B pathway
PPV	Positive Predictive Value
ROC	Receiver Operating Characteristic
SD	Standard Deviation
SE	Standard Error
SIRT1	Sirtuin 1
SP1	Specificity Protein 1 (transcription factor)
STAT3	Signal Transducer and Activator of Transcription 3
TIA	Transient Ischemic Attack
TOAST	Trial of Org 10172 in Acute Stroke Treatment
VEGF	Vascular Endothelial Growth Factor
